# Case report: Bronchoscopic intervention for rare benign airway tumors: a report of 4 cases and literature review

**DOI:** 10.3389/fonc.2024.1357982

**Published:** 2024-03-12

**Authors:** Zhifang Cui, Jinhong Wang, Hongwu Wang, Lei Li, Xiaohui Si, Yanbin Zhang, Heng Zou

**Affiliations:** ^1^ Department of Respiratory Medicine, Dongzhimen Hospital, Beijing University of Chinese Medicine, Beijing, China; ^2^ Department of Pulmonary and Critical Care Medicine, Sinopharm Tongmei General Hospital, Shanxi, China; ^3^ Department of Pathology, Dongzhimen Hospital, Beijing University of Chinese Medicine, Beijing, China

**Keywords:** bronchoscopic, endoscopic treatment, benign airway tumors, rare tumors, case series

## Abstract

Due to their unique location, airway tumors have a significant impact on patient quality of life and survival. Current research has focused extensively on malignant airway tumors; however, benign airway tumors, especially rare ones, are less understood due to their low incidence. These tumors are often misdiagnosed and mistreated due to diagnostic challenges. Therefore, there is still a lack of consensus on the treatment of some rare benign airway tumors. Our center summarizes the diagnosis and treatment of four rare cases of benign airway stenosis in recent years, highlighting the bronchoscopic manifestations and therapeutic approaches to improve the understanding of these diseases.

## 1 Introduction

Benign airway tumors are relatively rare, representing only 1.9% (1) of all airway tumors, with those in the tracheobronchial tract accounting for only 1% (2). Common types of benign airway tumors include hamartomas (chondromatous and non-chondromatous), inflammatory polyps, papillomas (simple, glandular, squamous), schwannomas, hemangiomas, and neurofibromas (3). Their primary clinical manifestations include airway obstruction-related symptoms, often misdiagnosed as bronchial asthma, chronic bronchitis, or malignancy. Delayed intervention can be life-threatening. Current treatments include surgical resection, lobectomy or pneumonectomy. Extensive resections for diffuse lesions have a significant impact on patient quality of life due to their invasiveness. In recent years, bronchoscopic techniques have shown advantages, including less trauma, faster postoperative recovery, fewer complications, and lower recurrence rates (4). Bronchoscopic treatments include high-frequency electrosurgery, argon plasma coagulation, and common thermal ablation methods such as laser (5). However, bronchoscopic tumor resection requires a high level of technical expertise, which limits its widespread use. Accurate intraoperative diagnosis and mastery of the surgical technique are crucial. For rare benign tracheobronchial tumors, the existing literature on bronchoscopic imaging and surgical methods is limited. We report 4 cases of rare benign tracheobronchial tumors. These cases presented with severe airway obstruction at diagnosis, with some lesions being diffuse, others highly vascular, and some almost completely obstructing the airway, posing significant surgical challenges. The primary treatment was bronchoscopic intervention, using rigid bronchoscopy for surgical safety, combined with both cryo and thermal ablation techniques. Current treatment outcomes are satisfactory, and hence this report is conducted.

## 2 Case presentation

### 2.1 Case 1

**Figure 1:**
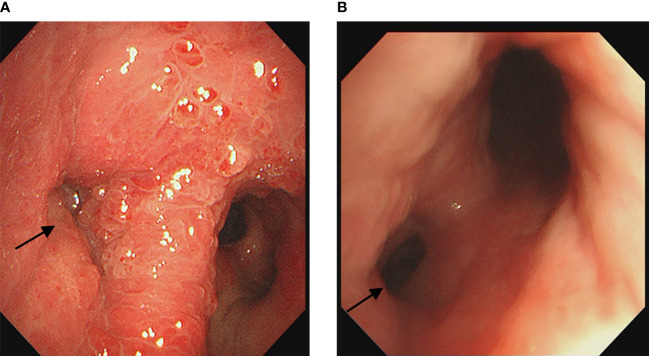
**(A)** Initial bronchoscopy showed near complete stenosis of the left main bronchus covered by scale-like neoplasms. **(B)** Follow-up bronchoscopy two months later showed approximately 50% stenosis of the left main bronchus and luminal patency of the right main bronchus. The arrows indicate the location of the lesion.

**Figure 2:**
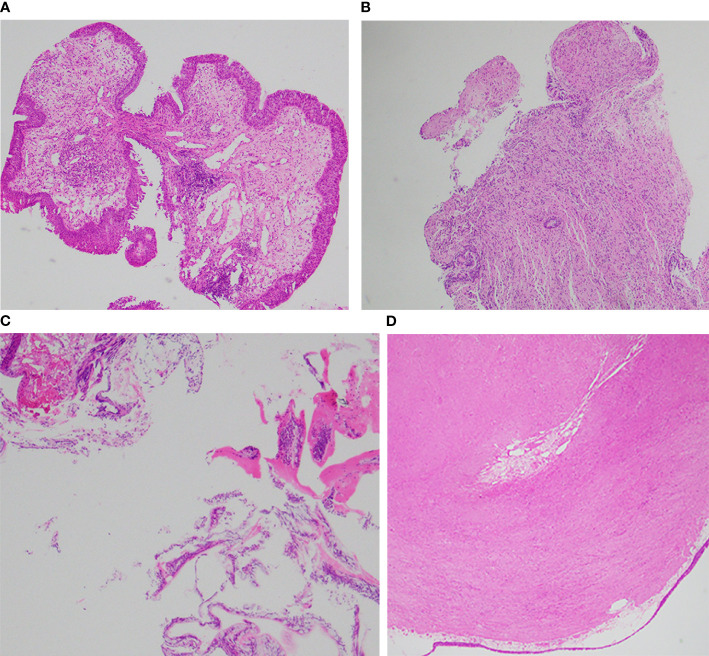
**(A)** Fibroepithelial polyp. **(B)** Granular cell tumor. **(C)** Mucous adenoma. **(D)** Leiomyoma.

A 60-year-old healthy male presented with intermittent cough and wheezing for four months. A CT scan from another hospital suggested obstructive atelectasis of the left upper lobe, raising the suspicion of lung cancer. Bronchoscopy and subsequent biopsy revealed bronchial mucosal hyperplasia and interstitial fibrous tissue proliferation without clear cancer components. Further chest CT at our hospital showed a mass at the left hilum with obstruction in the lower trachea and both main bronchi. Bronchoscopy showed scattered scale-like neoplasms near the carina, with the widened carina covered by scalelike neoplasms. The left main bronchus was almost completely obstructed by creeping scale-like neoplasms, making it impassable to the bronchoscope. The right main bronchus showed similar growth causing approximately 20% narrowing of the airway (**Figure 1A**). For larger lesions with clear boundaries, snare excision with an electric loop was performed at the base, followed by thermal resection. For smaller lesions or those with a creeping growth pattern, local argon plasma coagulation or carbon dioxide cryotherapy was used. Postoperative pathology revealed papillary and polypoid lesions covered with pseudostratified ciliated columnar epithelium, interstitial fibrosis, vascular proliferation with moderate inflammatory cell infiltration, and focal lymphoid follicle formation, leading to the diagnosis of fibroepithelial polyp (**Figure 2A**). The patient was treated with oral methylprednisolone (starting at 28mg Qd). A follow-up bronchoscopy two months later showed a patent airway near the carina and thickened carinal mucosa. The right main bronchus was clear, but the opening of the left main bronchus was narrowed by about 50% (**Figure 1B**). Multiple local injections of triamcinolone were administered at the area of narrowing. After seven months of follow-up, the patient had no significant coughing or wheezing symptoms.

### 2.2 Case 2

**Figure 3:**
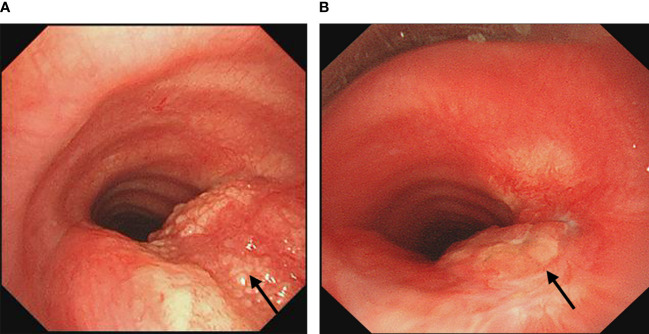
**(A)** Bronchoscopy showed a granular neoplasm in the right lower lateral wall of the main trachea, obstructing about 40% of the airway. **(B)** Bronchoscopy after eight months showed 15% airway stenosis at the original lesion site. The arrows indicate the location of the lesion.

A 54-year-old female presented with intermittent coughing for one year and was admitted to the hospital after the discovery of a tracheal mass 2 weeks earlier. The patient was initially diagnosed with an allergic cough due to her persistent coughing, and an external contrast-enhanced lung CT scan showed abnormal enhancement in the main bronchus. An external bronchoscopy revealed a mass in the main trachea. Postoperative pathology confirmed the presence of a granular cell tumor (**Figure 2B**). Surgical resection was recommended, but the patient refused. The patient finally underwent bronchoscopic tumor resection at our center. Bronchoscopy revealed a granular neoplasm on the right lower lateral wall of the main trachea. The tumor was irregular in shape, firm, tended to bleed on palpation, and obstructed approximately 40% of the airway (**Figure 3A**). Initially, laser thermal cutting and ablation were used, followed by high-frequency electrocautery at the junction of the lesion and mucosa. The lesion was then treated with carbon dioxide cryotherapy, snare excision, and biopsy forceps removal. After treatment, the airway stenosis was reduced to approximately 10%. A follow-up bronchoscopy two months later showed approximately 30% stenosis at the original lesion site. Larger lesions were treated again with laser and high-frequency electrocautery, while smaller post-treatment lesions were treated with snare excision, carbon dioxide cryotherapy, and biopsy forceps removal, reducing the stenosis to 10%. Eight months later, a follow-up bronchoscopy showed approximately 15% stenosis at the original lesion site (**Figure 3B**), which was reduced to approximately 10% after additional tumor resection. A follow-up CT scan after 12 months showed no significant stenosis, and no further bronchoscopic treatment was performed. The patient was followed for 14 months and has returned to a normal life with no significant clinical symptoms.

### 2.3 Case 3

**Figure 4:**
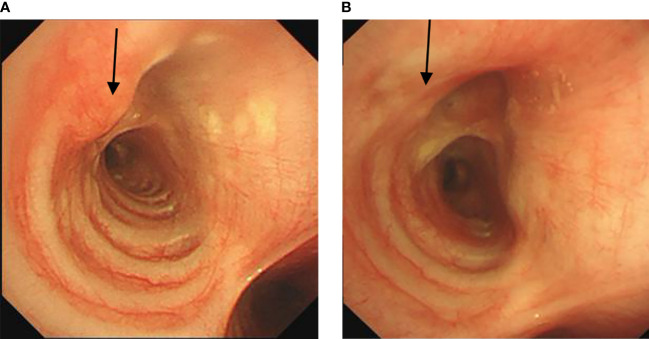
**(A)** Bronchoscopy showed a new neoplasm on the left upper wall of the opening of the right main bronchus, protruding into the airway. **(B)** Follow-up bronchoscopy six months later showed almost complete resolution of the neoplasm. The arrows indicate the location of the lesion.

A 16-year-old female student was admitted to the hospital complaining of intermittent wheezing, cough, and blood-stained sputum for more than 1 year. An external chest CT scan suggested a foreign body at the opening of the right main bronchus. External bronchoscopy revealed a tumor at this site. Postoperative pathology revealed a clear cell tumor in the right main bronchus, consistent with a diagnosis of pulmonary mucous adenoma (**Figure 2C**). The patient subsequently visited our center, where a bronchoscopy revealed a neoplasm on the upper left wall at the opening of the right main bronchus. The neoplasm protruded into the lumen in a pattern typical of a wall-based growth. Under endobronchial ultrasound, the neoplasm showed rich blood flow (**Figure 4A**). The lesion was resected with an electric snare at its base, and residual areas were treated with multiple cryoablations using a carbon dioxide cryotip. After treatment, the patient was free of wheezing, cough, and hemoptysis. A follow-up bronchoscopy six months later showed mild mucosal thickening and elevation at the original lesion site in the right main bronchus, which was treated with multiple carbon dioxide cryoablations (**Figure 4B**). The patient has been followed for 15 months, with no recent significant cough or wheezing symptoms.

### 2.4 Case 4

**Figure 5:**
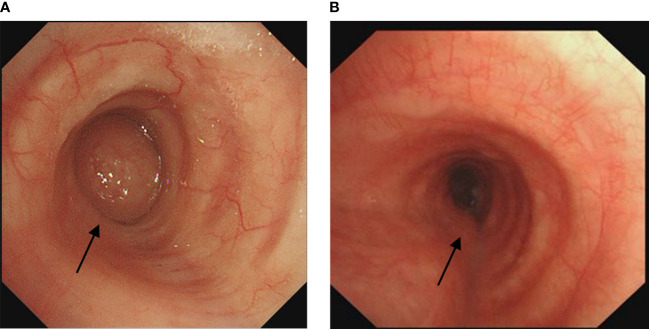
**(A)** Bronchoscopy showed a spherical tumor at the distal end of the left main bronchus, almost completely obstructing the airway.**(B)** Follow-up bronchoscopy 12 months later showed a patent distal end of the left main bronchus. The arrows indicate the location of the lesion.

A 45-year-old female was admitted to our hospital with complaints of a bronchial mass for 5 months. She had a history of dyspnea on exertion, and a routine physical examination with chest CT revealed an occupying lesion from the left main bronchus to the proximal lower lobe bronchus and atelectasis of the left lower lobe. Subsequent bronchoscopy at our center revealed a spherical tumor at the distal end of the left main bronchus. The tumor, with a surface showing tortuous blood vessels, was of intraluminal + wall-invading type, completely obstructing the left main bronchus (**Figure 5A**). An electric loop snare was applied at the base of the tumor for thermal excision, followed by the use of a carbon dioxide cryoprobe to freeze and extract the excised tissue. Bleeding during the procedure was controlled with argon plasma coagulation. Post-treatment observation showed that the tumor originated from the opening of the dorsal segment of the left lower lobe. The opening was visible, but the branches of the bronchus were completely obstructed by the tumor. However, the basal segment of the left lower lobe remained patent. Postoperative pathology revealed a leiomyoma at the opening of the left main bronchus (**Figure 2D**). The patient’s dyspnea was significantly relieved after surgery, and a follow-up chest CT showed a marked improvement in atelectasis of the left lower lung. Subsequent bronchoscopic examinations at our center at 3, 6, and 12 months after treatment showed a patent distal end of the left main bronchus with local mucosal elevation and scarring. The dorsal segment opening was not visible, but the basal segment opening was patent (**Figure 5B**). Multiple biopsies at the site of mucosal elevation in the distal left main bronchus consistently showed chronic membrane inflammation with fibrous and smooth muscle tissue proliferation. The patient has been followed for 22 months and is currently stable and able to live and work normally.

## 3 Discussion

Benign airway tumors are rare and often present clinically as symptoms of airflow obstruction. They can be easily confused with conditions such as bronchial asthma, bronchitis, or malignant tumors. Especially, rare airway tumors, which are rarely seen in clinical practice, are frequently misdiagnosed due to a lack of knowledge among physicians. Unclear diagnoses lead to unfocused treatment approaches, often resulting in poor prognosis and, in severe cases, life-threatening situations. Even when the diagnosis is clear, the traditional treatment largely relies on surgical resection, which is associated with high risk, significant trauma and poor patient compliance. The four cases in this report all presented with severe respiratory symptoms caused by airway obstruction and experienced diagnostic challenges. Some cases were even initially misdiagnosed as malignant tumors. These four patients, who were either ineligible for or refused surgical resection, presented with intraluminal lesions, meeting the criteria for treatment under bronchoscopy. After a thorough preoperative assessment at our center, they ultimately opted for bronchoscopic surgery. This paper reports the four cases of different rare benign airway tumors and discusses the bronchoscopic treatment and its efficacy, which is uncommon in the existing literature.

### 3.1 Tracheal fibroepithelial polyp

Fibroepithelial polyps are rare and benign, with a very low occurrence rate. While these polyps are most frequently found in the skin and urogenital tract (6), their presence in the respiratory tract is notably rare. To date, there are fewer than 30 publications on this topic, with the earliest known discovery and report by Rowlands in 1960 (7). The etiology of this condition is not fully understood, but is often speculated to be related to chronic airway inflammation. It is more common in males than in females (8). Lesions vary in size, with reported diameters ranging from 2 mm to 20 mm and an average size of 6.5 mm in existing studies (8). Clinical presentations vary in severity; smaller lesions are often found on physical examination, while larger lesions may present with symptoms such as hemoptysis, fever, and airway obstruction (9, 10). On bronchoscopy, they appear as round, polypoid lesions with a smooth surface, either circular or lobulated. The patient had extensive lesions. To avoid bleeding and improve surgical efficiency, for most of the lesions, we used an electric loop snare to encircle and thermally excise their bases, thereby enhancing efficiency while minimizing bleeding to the greatest extent. However, for smaller lesions and those with a creeping growth pattern, where the electric loop snare could not leverage its advantages, we employed argon plasma coagulation for localized thermal ablation and carbon dioxide cryoextraction for excision, both methods offered high precision. To our knowledge, there are no reports describing the bronchoscopic appearance of this condition. In our case, a white serous substance was observed oozing out under bronchoscopy after hot resection, with each treatment covering a small area. However, cryotherapy has shown better results in larger lesions with little bleeding. Initially, we suspected airway papilloma in this case. However, due to the lack of evidence of HPV infection, the combination of pathology consultations within and outside our hospital confirmed the diagnosis of fibroepithelial polyp. Due to its rarity, there is no clearly defined treatment approach for this condition in the literature. In our case, the patient had a diffuse area of lesions, making surgical resection less feasible. Most previously reported cases involve solitary lesions, with diffuse lesions being less common. Based on our center’s experience, diffuse lesions may be treated preferentially with bronchoscopic intervention, supplemented by a combination of steroids and antibiotics to improve efficacy. For solitary, large lesions or those that are difficult to remove under bronchoscopy, lobectomy may be necessary when indicated (11). For this condition, follow-up is recommended with bronchoscopy and chest CT scans. No recurrence has been observed during the short-term follow-up (3).

### 3.2 Tracheal granular cell tumor

Granular cell tumors are a type of benign tumor commonly found in the skin, gastrointestinal tract, and breast. Granular cell tumors have been reported in almost every part of the body, with an incidence rate of 0.03% (12). However, reports of these tumors in the airway are even rarer (13). These tumors grow slowly and the vast majority are benign, with malignant cases being rare (14). To date, no malignant cases have been reported in the trachea (15). The incidence of this disease is higher in females than in males (16, 17), and the specific mechanism of onset remains unclear. Tumor sizes range from 2 to 70 mm, with an average size of 11.44 mm (17). Approximately 73% of airway granular cell tumors are intraluminal, with others exhibiting both intraluminal and extraluminal characteristics (18). In the early stages of the disease, patients often have no clinical symptoms, but in later stages they may present with cough, hemoptysis, wheezing, and airway obstruction. Under bronchoscopy, these tumors appear as polypoid lesions, either pedunculated or non-pedunculated, with intact mucosa. Traditional bronchodilators and steroid hormones are largely ineffective in treating these tumors (4), and they are generally unresponsive to radiotherapy and chemotherapy (19). In our case, we observed that the granular cells formed a bead-like growth protruding into the lumen with invasion of the airway mucosa. The tumor was hard in consistency, making conventional carbon dioxide cryotherapy difficult due to low tissue yield. Thermal ablation showed advantages, but due to the hardness of the tumor, the debulking process was more challenging compared to other tumors. Due to the high energy and strong penetration capability of laser treatment, which can rapidly coagulate and vaporize tissues, we first used a laser to continuously ablate within the tumor to reduce its size. Subsequently, we used high-frequency electrocautery to spiral-cut the tissue along the airway wall, separating the tumor from the tracheal wall. Then, an electric loop snare was used to encircle and thermally cut the base of the tumor. For tissues not completely excised by thermal ablation and remaining on the tracheal wall, cryotherapy was employed to freeze and remove the mass to the greatest extent, ultimately achieving an ideal surgical outcome. At the first follow-up, we used low-temperature plasma technology on the residual tumor, and the clinical efficacy was also satisfactory. This approach could be considered for further studies. Follow-up with bronchoscopy and chest CT is recommended, as the recurrence rate is low (17).

### 3.3 Tracheal mucous adenoma

Mucous adenomas are rare benign tumors arising from salivary glands. Reports of this condition are scarce, with most of them concentrated before the year 2000, and currently, there are over 20 case reports (20–23). Existing studies have found that the incidence rate is higher in females than in males (24). These tumors most commonly occur in the trachea and bronchi, and occasionally in the lung (23). The average size of these tumors is about 17mm. It is relatively rare to find mucous adenomas larger than 30mm (21, 24). The cause of the disease remains unknown. Clinical manifestations vary depending on the location and size of the tumor. Under bronchoscopy, the tumor appears as a well-demarcated, shiny, hard mass, which may be pedunculated (25). Treatment generally involves surgery. Depending on the location and anatomic structure, options include lobectomy, segmentectomy, or wedge resection (22, 26). There are reports of endoscopic resection of the lesion (24, 25, 27), but most literature still recommends surgical excision as the standard treatment. In our case, the patient was young and the tumor was located in the right main bronchus. Surgical resection would have resulted in a significant loss of lung tissue and a high risk of complications. Therefore, we opted for thermal ablation using an electric loop snare at the base of the lesion under bronchoscopy. This proved to be effective, but the risk of intraoperative bleeding had to be considered. During surgery, ultrasound bronchoscopy revealed rich blood flow signals in the lesion, prompting the use of rigid bronchoscopy. First, electric loop snare excision and thermal ablation technology were employed to ensure safety. For the residual lesion parts after snare excision, carbon dioxide cryotherapy was adopted to freeze and melt the lesion tissue, thereby killing tumor cells and preventing regeneration. To date, no tumor recurrence has been reported in the literature (21, 22, 24, 26).

### 3.4 Tracheal leiomyoma

Leiomyomas are rare tumors of the airway. Literature reports that they account for approximately 1% of airway tumors and are more common in patients with compromised immune systems (28, 29). These tumors are more common in males than females, with a median age at diagnosis of 40.6 years (30). Respiratory tract leiomyomas can be classified based on their location into two types: pulmonary parenchymal and tracheobronchial. About 45% of respiratory tract leiomyomas are found within the bronchi (31). Those located in the pulmonary parenchyma often have no clinical symptoms, whereas tracheobronchial leiomyomas can cause symptoms related to airway obstruction, such as coughing, shortness of breath, wheezing, and in some cases, hemoptysis (32). Treatment options for tracheal leiomyoma include surgical resection, such as partial lobectomy or pneumonectomy, and bronchoscopic interventions, such as electrocautery, cryotherapy, and laser treatment. However, there is still controversy regarding the best approach. Some scholars argue that bronchoscopic interventions may not completely remove the tumor and may lead to recurrence, so surgical treatment is recommended (33). In our case, the tumor almost completely obstructed the airway, which posed a high risk of uncontrollable bleeding and involvement of the opposite lung lobe, making surgical intervention very risky. Respecting the patient’s wishes, we opted for bronchoscopic resection. Preoperative contingency plans were established, including the use of a rigid bronchoscope, maintaining the patient in the left lateral position, and pre-placement of a balloon catheter. During the surgery, electric loop snare excision and thermal ablation were employed to improve surgical efficiency and reduce intraoperative bleeding. For the tissue remaining in the lumen after thermal ablation, carbon dioxide cryotherapy was employed to freeze and remove the residual tissue from the airway smoothly.

The procedure went smoothly with controllable bleeding, and the bleeding site was cauterized with an argon plasma coagulation. This patient, who was treated with bronchoscopy, retained maximum anatomical structure and showed significant improvement in clinical symptoms. Currently, there is no evidence of recurrence. For leiomyomas with a simple intraluminal narrow base, bronchoscopic treatment is preferred because of its safety and efficacy (33, 34). For patients with both intraluminal and extraluminal lesions, surgical resection is the first choice when feasible. For patients unable to undergo thoracic surgery, bronchoscopic intraluminal treatment is an alternative, but close follow-up is required. The prognosis for this disease is generally good and recurrences are rare (34, 35).

## 4 Conclusion

Benign airway tumors are rare, and some of the rarer types are particularly prone to misdiagnosis due to their low incidence and lack of clinical experience. Clinicians often make a diagnosis based on patient symptoms, imaging findings, bronchoscopic appearance, and histologic features. Historically, the majority of treatments for benign airway tumors have been surgical resections, which are invasive with a high risk of complications and can significantly impact patient quality of life. However, with the advancement of bronchoscopic techniques in recent years, an increasing number of benign tumors in the trachea and bronchi are being treated by bronchoscopic surgery. Our center reported four cases of such rare benign airway tumors, all of which were primarily treated by bronchoscopic surgery. We meticulously planned each surgical procedure, discussed anesthesia risks with the anesthesia team, and jointly minimized surgical risks. The results of these surgeries have been quite favorable, and we are currently continuing follow-up. In this article, we have detailed the bronchoscopic surgical methods and obtained valuable bronchoscopic images of the lesions, contributing to the clinical experience in diagnosing and treating rare benign airway tumors. However, due to the limited number of cases, the individualized nature of these treatments, and the high technical and teamwork demands of bronchoscopy, the generalizability of our approach is somewhat limited. In the future, as the accuracy of diagnosis and treatment of rare benign airway tumors improves and bronchoscopic techniques become more widespread, we expect the number of patients undergoing bronchoscopic surgery to increase. This will allow cohort studies to further explore the advantages and disadvantages of bronchoscopic surgery compared to traditional surgical methods for the treatment of benign airway tumors.

## Data availability statement

The original contributions presented in the study are included in the article/supplementary material. Further inquiries can be directed to the corresponding author.

## Ethics statement

The studies involving humans were approved by Ethics Committee of Dongzhimen Hospital Affiliated to Beijing University of Chinese Medicine. The studies were conducted in accordance with the local legislation and institutional requirements. The participants provided their written informed consent to participate in this study. Written informed consent was obtained from the individual(s) for the publication of any potentially identifiable images or data included in this article. Written informed consent was obtained from the participant/patient(s) for the publication of this case report.

## Author contributions

ZC: Conceptualization, Formal analysis, Investigation, Writing – original draft. JW: Conceptualization, Formal analysis, Investigation, Writing – original draft. HW: Conceptualization, Methodology, Writing – review & editing. LL: Conceptualization, Resources, Writing – review & editing. XS: Conceptualization, Resources, Writing – review & editing. YZ: Conceptualization, Resources, Writing – review & editing. HZ: Conceptualization, Supervision, Writing – review & editing.

## References

[1] Shah H Garbe L Nussbaum E Dumon JF Chiodera PL Cavaliere S. Benign tumors of the tracheobronchial tree. Endoscopic characteristics and role of laser resection. Chest. (1995) 107:1744–51. doi: 10.1378/chest.107.6.1744 7781378

[2] Hurt R. Benign tumours of the bronchus and trachea, 1951-1981. Ann R Coll Surg Engl. (1984) 66:22–6. PMC2493627 6362537

[3] Labarca G Caviedes I Vial MR Pires Y Folch E Majid A. Airway fibroepithelial polyposis. Respir Med Case Rep. (2017) 22:154–7. doi: 10.1016/j.rmcr.2017.08.005 PMC5554959 28831375

[4] Ipakchi R Zager WH de Baca ME Bloedon E McCue PA Zwillenberg D. Granular cell tumor of the trachea in pregnancy: A case report and review of literature. Laryngoscope. (2004) 114:143–7. doi: 10.1097/00005537-200401000-00026 14710011

[5] Guarnieri T Cardinale L Macchia G Cortese G Veltri A. Multiphasic multidetector computed tomography study of a rare tracheal tumor: granular cell tumor. Case Rep Pulmonol. (2014) 2014:807430. doi: 10.1155/2014/807430 25548708 PMC4274649

[6] Nowak MA Marzich CS Scheetz KL McElroy JB. Benign fibroepithelial polyps of the renal pelvis. Arch Pathol Lab Med. (1999) 123:850–2. doi: 10.5858/1999-123-0850-BFPOTR 10458839

[7] Rowlands DT Jr. Fibroepithelial polyps of the bronchus: A case report and review of the literature. Dis Chest. (1960) 37:199–202. doi: 10.1378/chest.37.2.199 14439715

[8] Casalini E Cavazza A Andreani A Marchioni A Montanari G Cappiello FG. Bronchial fibroepithelial polyp: A clinico-radiologic, bronchoscopic, histopathological and *in-situ* hybridisation study of 15 cases of a poorly recognised lesion. Clin Respir J. (2017) 11:43–8. doi: 10.1111/crj.12300 25832329

[9] Georgakopoulou VE Kourtelesi E Mermigkis D Trakas N Tsiafaki X. Bronchial fibroepithelial polyp with severe hemoptysis as first manifestation: A case report. Cureus. (2020) 12:e10261. doi: 10.7759/cureus.10261 33042699 PMC7536118

[10] Abhinav Agrawal AA Alagusundarmoorthy SS Meena N. Benign endobronchial neoplasms: A review. J Pulmonary Respir Med. (2015) 05:1–7. doi: 10.4172/2161-105x.1000275

[11] Ushiki A Yasuo M Tanabe T Urushihata K Yamamoto H Hanaoka M. A rare case of a tracheal fibroepithelial polyp treated by an endobronchial resection. Intern Med. (2008) 47:1723–6. doi: 10.2169/internalmedicine.47.1241 18827424

[12] Lack EE Worsham GF Callihan MD Crawford BE Klappenbach S Rowden G. Granular cell tumor: A clinicopathologic study of 110 patients. J Surg Oncol. (1980) 13:301–16. doi: 10.1002/jso.2930130405 6246310

[13] Amar YG Nguyen LH Manoukian JJ Nguyen VH O'Gorman A Shapiro R. Granular cell tumor of the trachea in a child. Int J Pediatr Otorhinolaryngol. (2002) 62:75–80. doi: 10.1016/s0165-5876(01)00599-7 11738699

[14] Leboulanger N Rouillon I Papon JF Josset P Roger G Garabédian EN. Childhood granular cell tumors: two case reports. Int J Pediatr Otorhinolaryngol. (2008) 72:279–83. doi: 10.1016/j.ijporl.2007.10.021 18082899

[15] Ordóñez NG. Granular cell tumor: A review and update. Adv Anat Pathol. (1999) 6:186–203. doi: 10.1097/00125480-199907000-00002 10410172

[16] Rizzo A Serban ED Ricci AD Nannini M Saponara M Cancellieri A. Granular cell tumor of the trachea as a rare cause of dyspnea in a young woman. Respir Med Case Rep. (2019) 28:100961. doi: 10.1016/j.rmcr.2019.100961 PMC6838894 31720208

[17] Mobarki M Dumollard JM Dal Col P Camy F Peoc'h M Karpathiou G. Granular cell tumor a study of 42 cases and systemic review of the literature. Pathol Res Pract. (2020) 216:152865. doi: 10.1016/j.prp.2020.152865 32089415

[18] Desai DP Maddalozzo J Holinger LD. Granular cell tumor of the trachea. Otolaryngol–Head Neck Surg. (1996) 120:595–8. FACS, FAAP, Chicago, Illinois. doi: 10.1053/hn.1999.v120.a84488 10187970

[19] Joung MK Lee YJ Chung CU Lee JE Jung SS Kim SY. A case of granular cell tumor of the trachea. Korean J Intern Med. (2007) 22:101–5. doi: 10.3904/kjim.2007.22.2.101 PMC2687619 17616025

[20] Sasaki E Masago K Kogure Y Fujita S Iwakoshi A Kuroda H. Mucous gland adenoma of the lung: A neoplastic counterpart of mucinous bronchial glands. Mod Pathol. (2023) 36:100182. doi: 10.1016/j.modpat.2023.100182 37028599

[21] Zaleski MP Kalhor N Moran CA. Mucous gland adenoma: the spectrum of growth patterns and the diagnostic challenges. Adv Anat Pathol. (2020) 27:371–9. doi: 10.1097/PAP.0000000000000283 32909967

[22] Uluşan A. A rare case of peripherally located non-bronchial pulmonary mucous gland adenoma. Turkish J Thorac Cardiovasc Surg. (2018) 26:664–7. doi: 10.5606/tgkdc.dergisi.2018.15357 PMC7018175 32082814

[23] Lee B Choi IH Han J Lee KS Shim YM. An unusual case of pulmonary mucous gland adenoma with fibromyxoid stroma and cartilage islands in 68-year-old woman. Korean J Pathol. (2014) 48(2):167–9. doi: 10.4132/KoreanJPathol.2014.48.2.167 PMC4026811 24868233

[24] Tauziede-Espariat A Grand B Georges O Benali A Viehl P Bitton L. A case of bronchial mucous gland adenoma: A rare diagnosis that should not be mistaken! Ann Pathol. (2021) 41:192–5. doi: 10.1016/j.annpat.2020.11.008 33390273

[25] Couraud S Isaac S Guibert B Souquet PJ. Bronchial mucous gland adenoma revealed following acute pneumonia. Interactive Cardiovasc Thorac Surg. (2011) 14:347–9. doi: 10.1093/icvts/ivr104 PMC3290366 22186127

[26] Zhang XT Yang M Liu XF Lin XY. Peripheral mucous gland adenoma of the lung with parenchymal involvement and smooth muscle in the stroma: A rare case report and literature review. Med (Baltimore). (2018) 97:e9597. doi: 10.1097/MD.0000000000009597 PMC5779752 29504983

[27] Vergnenègre C Hureaux J Morvant B Urban T Jeanfaivre T. Un adénome des glandes muqueuses bronchiques réséqué Par voie endoscopique. Rev Des Maladies Respiratoires. (2017) 34:253–6. doi: 10.1016/j.rmr.2016.07.008 28341128

[28] Lee ES Locker J Nalesnik M Reyes J Jaffe R Alashari M. The association of epstein-barr virus with smooth-muscle tumors occurring after organ transplantation. N Engl J Med. (1995) 332:19–25. doi: 10.1056/nejm199501053320104 7990861

[29] Zhang Y Ma H. The right apical tracheal bronchus with bronchial leiomyoma. Medicine. (2022) 101(10):e29181. doi: 10.1097/md.0000000000029181 35475803 PMC9276463

[30] White SH Ibrahim NB Forrester-Wood CP Jeyasingham K. Leiomyomas of the lower respiratory tract. Thorax. (1985) 40:306–11. doi: 10.1136/thx.40.4.306 PMC460053 4023981

[31] Wilson RW Kirejczyk W. Pathological and radiological correlation of endobronchial neoplasms: part I, benign tumors. Ann Diagn Pathol. (1997) 1:31–46. doi: 10.1016/S1092-9134(97)80007-X 9869824

[32] Ambi U Hosalli V Ganeshnavar A Hulakund S Prakashappa DS. Anaesthetic considerations in primary repair of tracheobronchial injury following blunt chest trauma in paediatric age group: experience of two cases. Indian J Anaesthesia. (2013) 57(4):410–2. doi: 10.4103/0019-5049.118541 PMC3800340 24163462

[33] Park JS Lee M Kim HK Choi YS Kim K Kim J. Primary leiomyoma of the trachea, bronchus, and pulmonary parenchyma – a single-institutional experience. Eur J Cardio-Thoracic Surg. (2011) 41:41–5. doi: 10.1016/j.ejcts.2011.03.051 PMC3241106 21767960

[34] Kwon YS Kim H Koh WJ Suh GY Chung MP Kwon OJ. Clinical characteristics and efficacy of bronchoscopic intervention for tracheobronchial leiomyoma. Respirology. (2008) 13:908–12. doi: 10.1111/j.1440-1843.2008.01366.x 18811890

[35] Rodríguez E Pombo F Aguilera C Capdevila A. Recurring tracheal leiomyoma presenting as a calcified mediastinal mass. Eur J Radiol. (1996) 23:82–4. doi: 10.1016/0720-048x(95)00718-6 8872076

